# Investigation of supramolecular synthons and structural characterisation of aminopyridine-carboxylic acid derivatives

**DOI:** 10.1186/1752-153X-8-31

**Published:** 2014-05-06

**Authors:** Madhukar Hemamalini, Wan-Sin Loh, Ching Kheng Quah, Hoong-Kun Fun

**Affiliations:** 1 X-ray Crystallography Unit, School of Physics, Universiti Sains Malaysia, 11800 USM Penang, Malaysia; 2 Department of Pharmaceutical Chemistry, College of Pharmacy, King Saud University, Riyadh 11451, Saudi Arabia

## Abstract

**Background:**

Co-crystal is a structurally homogeneous crystalline material that contains two or more neutral building blocks that are present in definite stoichiometric amounts. The main advantage of co-crystals is their ability to generate a variety of solid forms of a drug that have distinct physicochemical properties from the solid co-crystal components. In the present investigation, five co-crystals containing 2-amino-6-chloropyridine (AMPY) moiety were synthesized and characterized.

**Results:**

The crystal structure of 2-amino-6-chloropyridine (AMPY) (I), and the robustness of pyridine-acid supramolecular synthon were discussed in four stoichiometry co-crystals of AMPY…BA (II), AMPY…2ABA (III), AMPY…3CLBA (IV) and AMPY…4NBA (V). The abbreviated designations used are benzoic acid (BA), 2-aminobenzoic acid (2ABA), 3-chlorobenzoic acid (3CLBA) and 4-nitrobenzoic acid (4NBA). All the crystalline materials have been characterized by ^1^HNMR, ^13^CNMR, IR, photoluminescence, TEM analysis and X-ray diffraction. The supramolecular assembly of each co-crystal is analyzed and discussed.

**Conclusions:**

Extensive N**---**H · · · N/N**---**H · · · O/O**---**H · · · N hydrogen bonds are found in **(I-V),** featuring different supramolecular synthons. In the crystal structure, for compound **(I)**, the 2-amino-6-chloropyridine molecules are linked together into centrosymmetric dimers by hydrogen bonds to form homosynthon, whereas for compounds **(II-V)**, the carboxylic group of the respective acids (benzoic acid, 2-aminobenzoic acid, 3-chlorobenzoic acid and 4-nitrobenzoic acid) interacts with pyridine molecule in a linear fashion through a pair of N**---**H · · · O and O**---**H · · · N hydrogen bonds, generating cyclic hydrogen-bonded motifs with the graph-set notation

$$R_{2}^{2}\left(8\right)$$

, to form heterosynthon. In compound **(II)**, another intermolecular N**---**H · · · O hydrogen bonds further link these heterosynthons into zig-zag chains. Whereas in compounds **(IV)** and **(V)**, these heterosynthons are centrosymmetrically paired *via* N**---**H · · · O hydrogen bonds and each forms a complementary DADA [D = donor and A = acceptor] array of quadruple hydrogen bonds, with graph-set notation $R_{2}^{3}\left(8\right)$, $R_{2}^{2}\left(8\right)$ and $R_{2}^{3}\left(8\right)$.

## Background

Co-crystal is a structurally homogeneous crystalline material that contains two or more neutral building blocks that are present in definite stoichiometric amounts. The main advantage of co-crystals is their ability to generate a variety of solid forms of a drug that have distinct physicochemical properties from the solid co-crystal components. Such properties include, but are not limited to, solubility, dissolution, bioavailability, hygroscopicity, hydrate/solvate formation, crystal morphology, fusion properties, chemical and thermal stabilities, and mechanical properties. Understanding the knowledge of supramolecular synthons is important for hydrogen bond construction. There are two types of synthons which are supramolecular homosynthon (composed of self-complementary functional groups, as exemplified by the carboxylic acid dimer) and supramolecular heterosynthon [1] (composed of different but complementary functional groups). For instance, the latter includes acid…pyridine [2], acid…amide [3,4], hydroxyl…amine [5] and hydroxyl…pyridine [6] supramolecular synthons with typical distance ranges for these frequent supramolecular heterosynthons are ca. 2.5-2.8 Å, 2.4-2.8 Å, 2.5-3.0 Å, and 2.5-3.1 Å, respectively. The crucial use of 2-aminopyridine is as an intermediate in the manufacture of pharmaceuticals, particularly in anti-histamines and *piroxican. Lornoxican* and *Tenoxican* are considered as new non-steroidal and anti-inflammatory drugs of the oxicam class inhibiting cyclooxygenase which is the key enzyme of prostaglandin biosynthesis at the site of inflammation [7]. The aminopyridine–carboxylate/carboxylic acid systems may adopt two different proton-limiting structures, namely, O**---**H · · · N (1) → O^-^**---**H · · · N^+^(2), which yield hydrogen-bonding and ionic interactions, respectively. These two types of configurations can be represented by the graph-set designator $R_{2}^{2}\left(8\right)$[8]. This $R_{2}^{2}\left(8\right)$ motif [robust motif] has been observed in DHFR-TMP [2,4-diamino-5-(3′,4′,5′-trimethoxybenzylpyrimidine)] complexes [9] and it is one of the 24-most frequently observed cyclic-hydrogen bonded motifs in organic crystal structures [10]. The various hydrogen-bonding patterns involving aminopyrimidine–carboxylate interactions have been reported in the literatures [11]. Many of the recurring hydrogen-bonded motifs leading to supramolecular architectures play a significant role in crystal engineering [12,13]. The study of co-crystals is of sprouting interest since Active Pharmaceutical Ingredient (API) properties can be modified in a graded manner by revolving into co-crystals [14]. In the present investigation, we have chosen 2-amino-6-chloropyridine (AMPY) (its neutral form) **(I)**, because the molecules of this ligand are self-assembled *via* N**---**H · · · N hydrogen bonds to form homosynthon. It also interacts with carboxylic acid molecules through N**---**H · · · O hydrogen bonds, to form heterosynthon, and paired centrosymmetrically *via* another N**---**H · · · O hydrogen bonds, to form a DADA array by multiple hydrogen bonds. The later is a habitually occurring synthon which occurs in amine-carboxylic acid systems. The carboxylic acids referred to in this study, together with their abbreviated designations, are: benzoic acid (BA), 2-aminobenzoic acid (2ABA), 3-chlorobenzoic acid (3CLBA) and 4-nitrobenzoic acid (4NBA). The co-crystals were analyzed by IR spectroscopy, ^1^HNMR, ^13^CNMR, photoluminescence, TEM analysis and X-ray diffraction.

## Results and discussion

**Figure 1:**
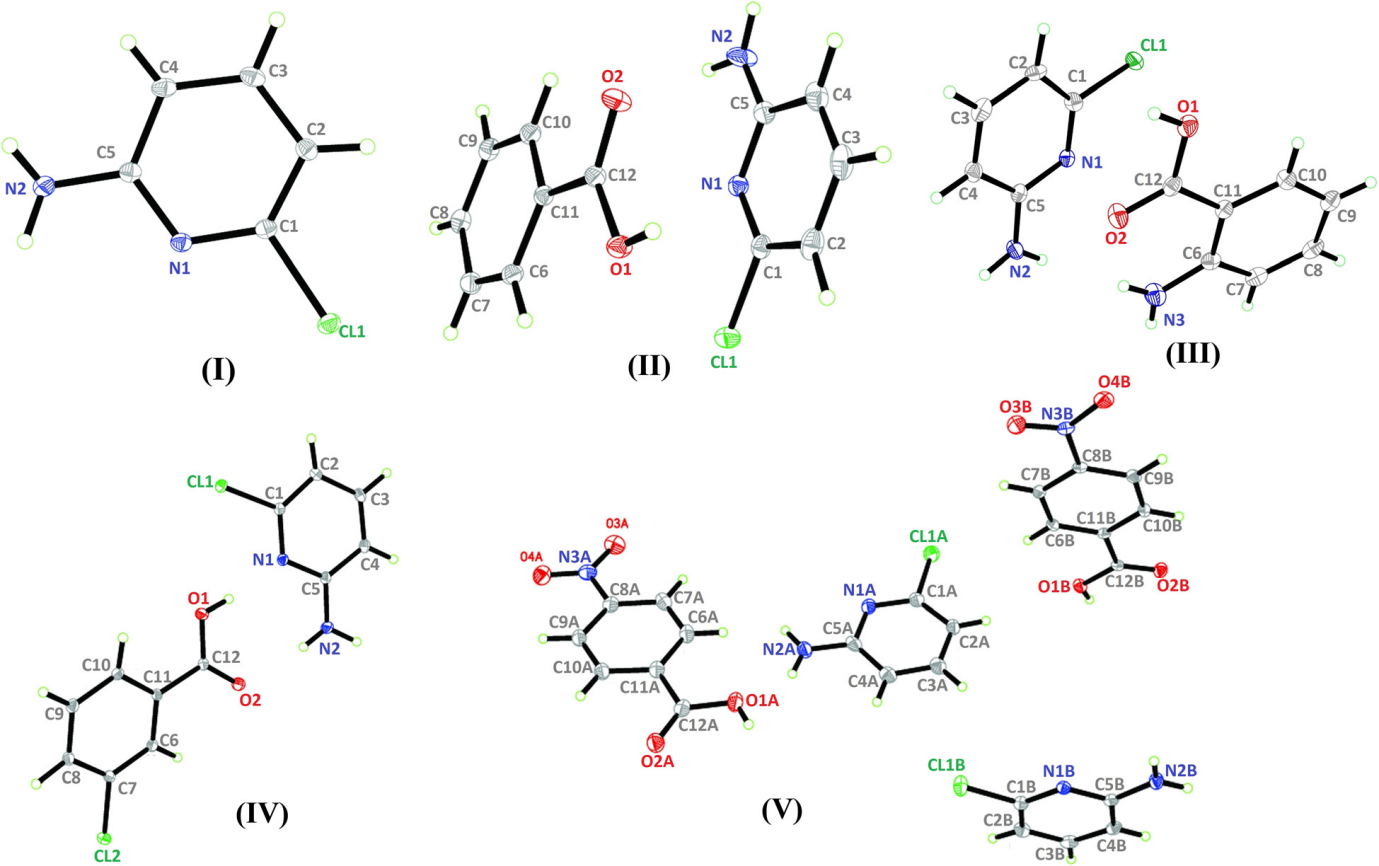
An ORTEP view of the compounds (I-V), showing 30% probability displacement ellipsoids.

The targeted molecules, AMPY **(I)**, AMPY…BA **(II)**, AMPY…2ABA (**III)**, AMPY…3CLBA **(IV)** and AMPY…4NBA **(V)**, were prepared and their crystal structures were determined. ORTEP views of these compounds **(I-V)** are shown in Figure 1. The crystal structures of **(I-V)** have been determined using single-crystal X-ray diffraction. Crystallographic data for compounds **(I-V)** are presented in Table 1, whereas hydrogen bond geometries are listed in Table 2. The purity of the solid phase of these complexes was characterized by XRPD. All the crystalline materials have been characterized by ^1^HNMR, ^13^CNMR, IR, photoluminescence and TEM analysis.

**Table 1:** Crystallographic data for compounds (I-V)

**Compound**	**I**	**II**	**III**	**IV**	**V**
CCDC deposition number	806013	806014	806010	806011	806012
Molecular formula	C_5_H_5_N_2_Cl	C_12_H_11_N_2_O_2_Cl	C_12_H_12_N_3_O_2_Cl	C_12_H_10_N_2_O_2_Cl	C_12_H_10_N_3_Cl
Molecular weight	128.56	250.68	265.70	285.12	295.68
Crystal system	Monoclinic	Orthorhombic	Monoclinic	Triclinic	Triclinic
Space group	*P*2_1_/*c*	*P*2_1_2_1_2_1_	*P*2_1_/*c*	Pbar-1	Pbar-1
*a* (Å)	11.9337 (10)	5.5003 (7)	17.061 (4)	3.7662 (4)	7.0854 (5)
*b* (Å)	4.6994 (4)	13.3896 (18)	5.3159 (15)	13.1822 (15)	7.3910 (5)
*c* (Å)	11.2252 (10)	16.314 (2)	13.700 (4)	13.1943 (15)	24.3724 (18)
α (°)	90	90	90	109.148 (2)	85.938 (1)
β (°)	112.601 (2)	90	104.642 (10)	92.991 (2)	82.727 (1)
γ (°)	90	90	90	96.214 (2)	86.869 (1)
*V (*Å^3^)	581.18 (9)	1201.5 (3)	1202.2 (6)	612.49 (12)	1261.54 (15)
*Z*	4	4	4	2	4
*D*_calc_ (g cm^-3^)	1.469	1.386	1.468	1.546	1.557
Crystal dimensions (mm)	0.33 × 0.28 × 0.15	0.51 × 0.11 × 0.06	0.43 × 0.16 × 0.04	0.58 × 0.15 × 0.03	0.47 × 0.08 × 0.05
Colour	Colourless	Colourless	Brown	Colourless	Yellow
μ (mm^-1^)	0.535	0.309	0.315	0.524	0.321
Radiation λ (Å)	0.71073	0.71073	0.71073	0.71073	0.71073
*T*_min_/*T*_max_	0.8444/0.9226	0.8577/0.9829	0.8756/0.9869	0.7509/0.9834	0.8647/0.9857
Reflections measured	7105	9878	12497	12709	22025
Ranges/indices (*h*, *k*, *l*)	-16, 16; -6, 6;	-8, 8; -16, 20;	-24, 23; -7, 7;	-5, 5; -18, 18;	-9, 9; -9, 8;
	-15, 15	-22, 24	-19, 19	-18, 18	-31, 31
θ limit (°)	1.9–30.0	2.9–32.6	2.5–30.1	1.6–30.0	1.7–27.5
Unique reflections	1670	4357	3500	3538	5750
Observed reflections	1531	3289	3007	2847	4709
(*I* > 2σ(*I*))					
Parameters	73	157	164	163	441
Goodness of fit on *F*^2^	1.099	0.98	1.12	1.04	0.98
*R*_1_, *wR*_2_ [*I* ≥ 2σ(*I*)]	0.033, 0.092	0.046, 0.105	0.089, 0.237	0.038, 0.107	0.054, 0.163

**Table 2:** Hydrogen-bond geometries for compounds (I-V)

***D***–**H · · ·** ***A***	***d*** **(*D***–**H) (Å)**	***d*** **(H · · ·** ***A*) (Å)**	***d*** **(*D*** **· · ·** ***A*) (Å)**	***Angle *** **(*D***–**H · · ·** ***A*) (°)**
**I**				
N2—H1N2 · · · N2^i^	0.83	2.55	3.326 (2)	155
N2—H2N2 · · · N1^ii^	0.90	2.22	3.112 (2)	173
C4—H4A · · · Cl1^iii^	0.93	2.83	3.623 (1)	144
**II**				
O1—H1N2 · · · O2	0.89	1.99	2.872 (2)	172
N2—H1N2 · · · O2^iv^	0.87	2.04	2.905 (2)	172
N2—H2N2 · · · O2	0.88 (3)	2.03 (3)	2.906 (2)	173 (2)
**III**				
N2—H1N2 · · · O2^v^	0.86	2.10	2.940 (6)	165
O1—H1O1 · · · N1^vi^	0.82	1.98	2.791 (7)	171
N3—H2N3 · · · O2	0.86	2.06	2.686 (6)	129
**IV**				
O1—H1O1 · · · N1	0.84	1.87	2.703 (2)	170
N2—H1N2 · · · O2^vii^	0.90	2.15 (2)	2.952 (2)	149
N2—H2N2 · · · O2	0.88	2.06	2.934 (2)	169
**V**				
N2—H2AB · · · O2A^viii^	0.86	2.12	2.963 (3)	167
N2A—H2AC · · · O2A^ix^	0.82	2.34	3.060 (4)	141
O1A—H1OA · · · N1A^x^	0.82	1.88	2.685 (3)	168
O1B—H1OB · · · N1B^viii^	0.82	1.88	2.684 (3)	169
C3A—H3AA · · · O3B^xi^	0.93	2.50	3.157 (4)	128

Symmetry codes: (i) = 1-*x*,-1/2 + *y*,1/2-*z*; (ii) = 1-*x*,-*y*,1-*z*; (iii) = *x*,-1/2-*y*,-1/2 + *z*; (iv) = 1/2 + *x*,3/2-*y*,2-*z*; (v) = -1 + *x*,*y*,*z*; (vi) = -1/2 + *x*,3/2-*y*,2-*z*; (vii) = 1-*x*,1-*y*,1-*z*; (viii) = *x*,-1 + *y*,*z*; (ix) = 1-*x*,2-*y*,1-*z*; (x) = *x*,1 + *y*,*z*; (xi) = 1 + *x*,*y*,*z.*

### X-ray crystallography

In all compounds (**I-V**), atoms in the pyridine ring are coplanar with maximum deviations of 0.005 (1) Å **(I)**, 0.004 (2) Å **(II)**, 0.009 (5) Å **(III)**, 0.008 (2) Å **(IV)** and 0.002 (3) Å (molecule A):0.007 (2) Å (molecule B) **(V)**, respectively. The C5-N2 [1.3674 (16) Å **(I)**, 1.340 (2) Å **(II)**, 1.360 (8) Å **(III)**, 1.354 (2) Å **(IV)** and 1.355 (4) Å (molecule A): 1.345 (4) Å (molecule B) **(V)**] bond lengths are approximately equal to that of a C = N double bond, indicating that atom N2 of the exo amine group must also be *sp*^2^ hybridized. This is further supported by the C5—N2—H1N2/H2N2 angles which are in the range of 115.1-120.0° and the fact that atoms C5, N2, H1N2 and H2N2 lie almost in the pyridine plane. Similar bond distances and angles have been observed in 2-aminopyridinium succinate-succinic acid [15]. Proton transfer does not take place from the carboxylic acid to the N atom of 2-amino-6-chloropyridine ring, and the internal C1—N1—C5 angles are 116.8 (1)° **(I)**, 117.1 (2)° **(II)**, 116. 5 (4)° **(III)**, 117.1 (1)° **(IV)** and 117.1 (3)° (molecule A):117.3 (2)° (molecule B) **(V)**. Compound **(III)** is a non-merohedral twin with the refined ratio of twin components being 0.276 (6):0.724(6). In **(IV)**, a significant structural change in 3-chlorobenzoic acid has been observed with C-Cl (1.748 (2) Å) and C = O (1.220 (2) Å) bonds which have adopted a cisoid conformation that differ from the pure 3-chlorobenzoic acid which is in a transoid conformation [16]. In **(V)**, the nitro group of the 4-nitrobenzoic acid molecule is twisted slightly from the attached ring and the dihedral angles between N1/C1—C5 and O3—O4/C3/N1 planes are 5.91 (16)° (molecule A) and 5.79 (15)° (molecule B).

### Hydrogen bonding

**Scheme 1:**
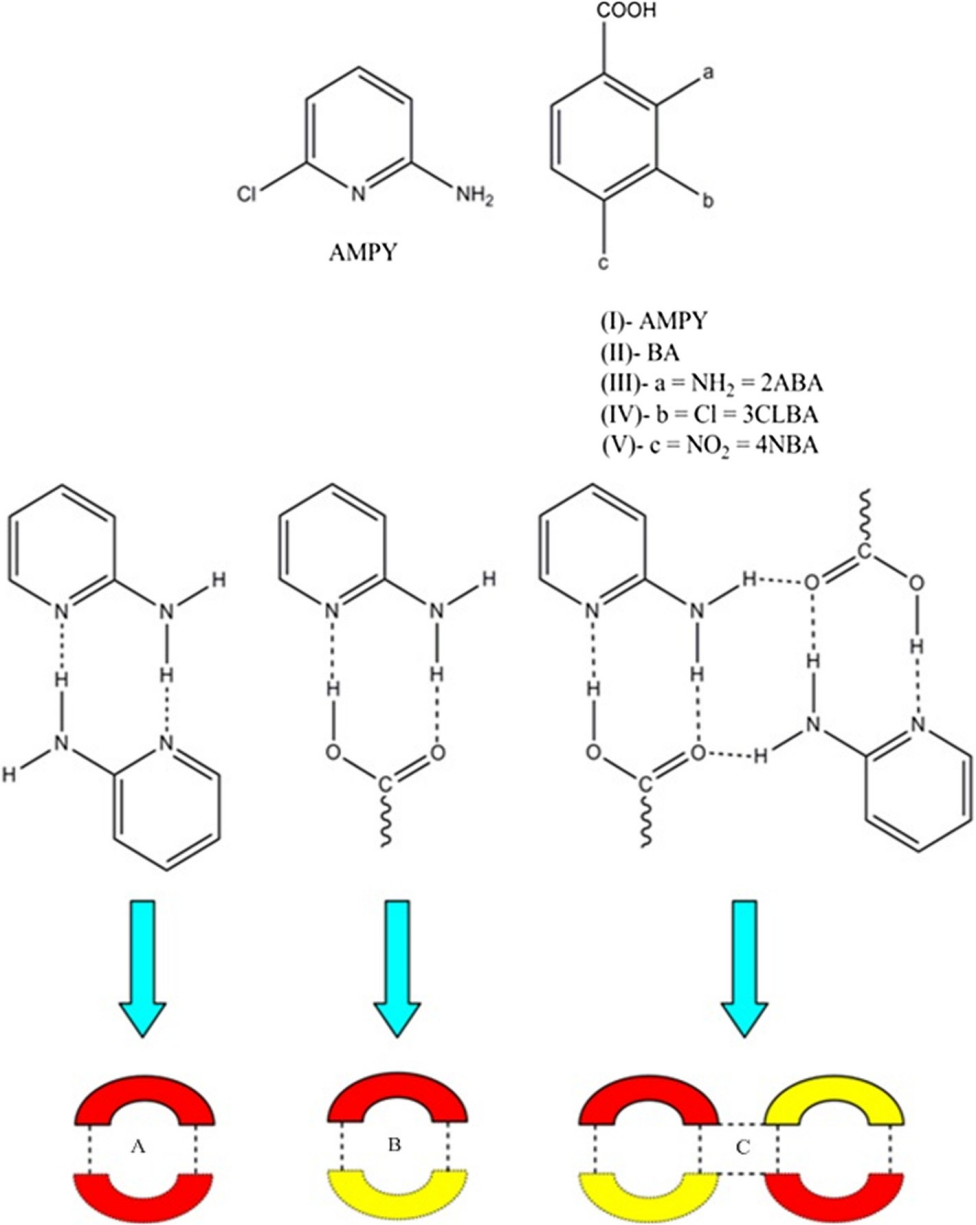
**Three types of supramolecular synthons, (A), (B) and (C), were discussed in this paper. (A)** & **(B)** represent homo and heterosynthons, $R_{2}^{2}\left(8\right)$, respectively whereas **(C)** represents a DADA array, $R_{2}^{3}\left(8\right)$: $R_{2}^{2}\left(8\right)$: $R_{2}^{3}\left(8\right)$.

**Figure 2:**
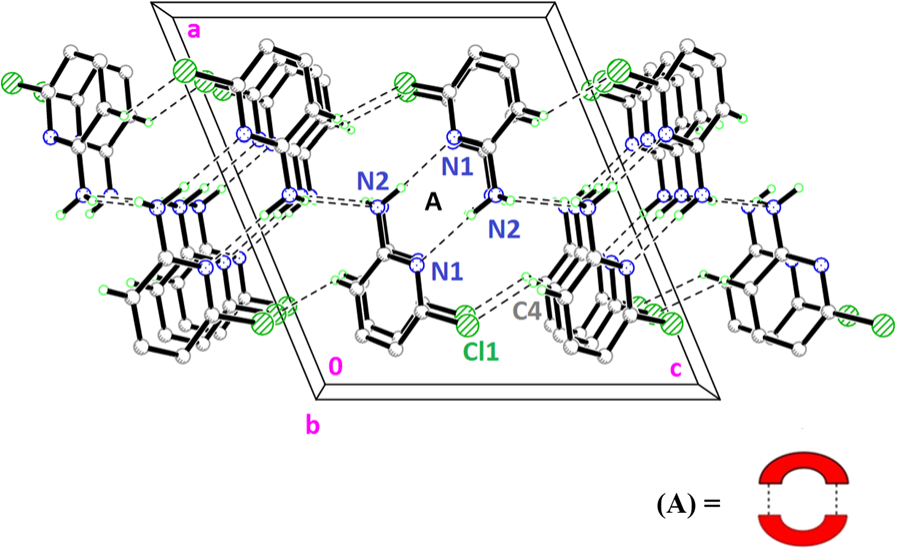
Hydrogen bonding patterns in compound (I), (A) represents homosynthon.

**Figure 3:**
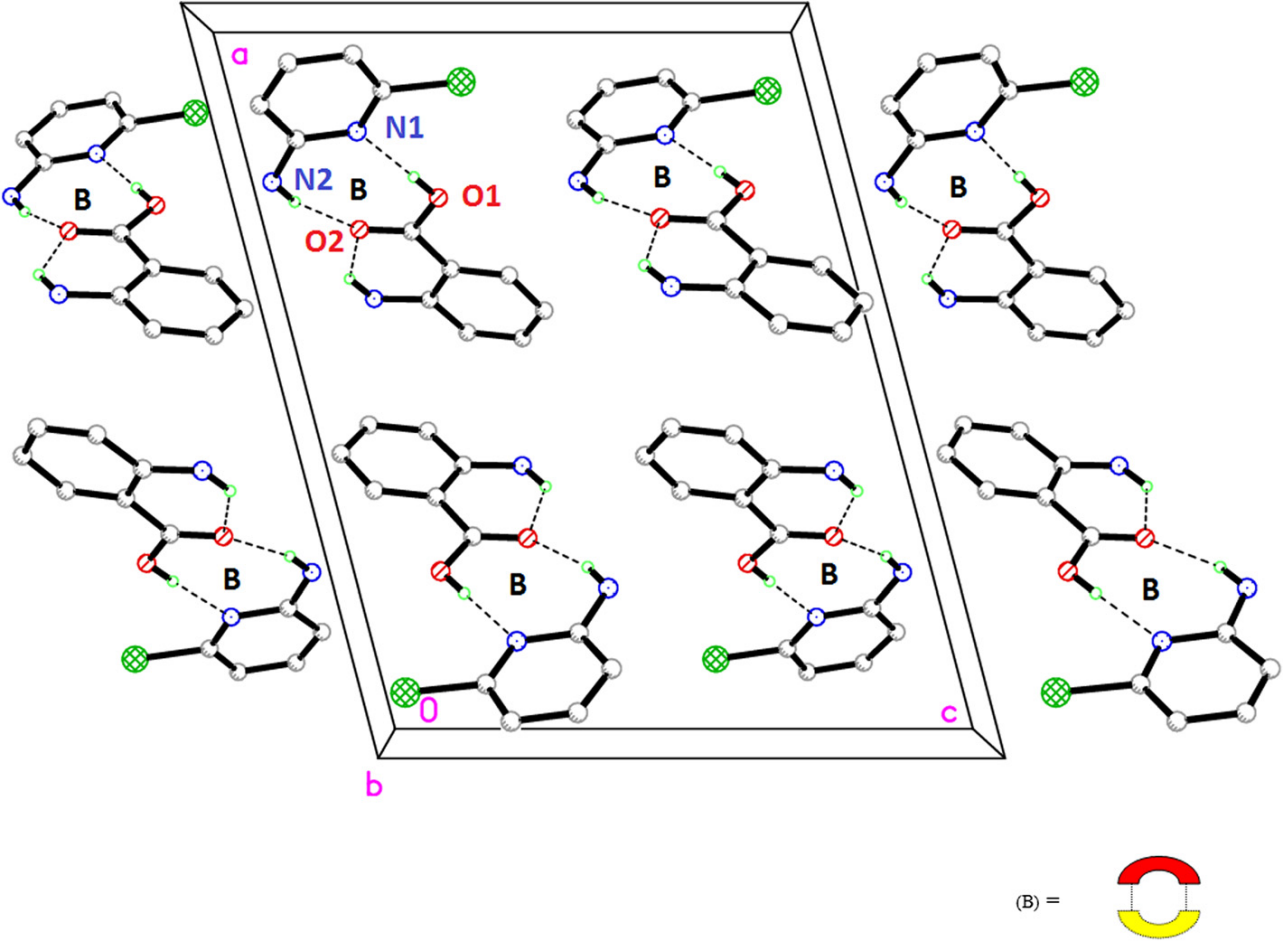
Hydrogen bonding patterns in compound (III), (B) represents heterosynthon.

**Figure 4:**
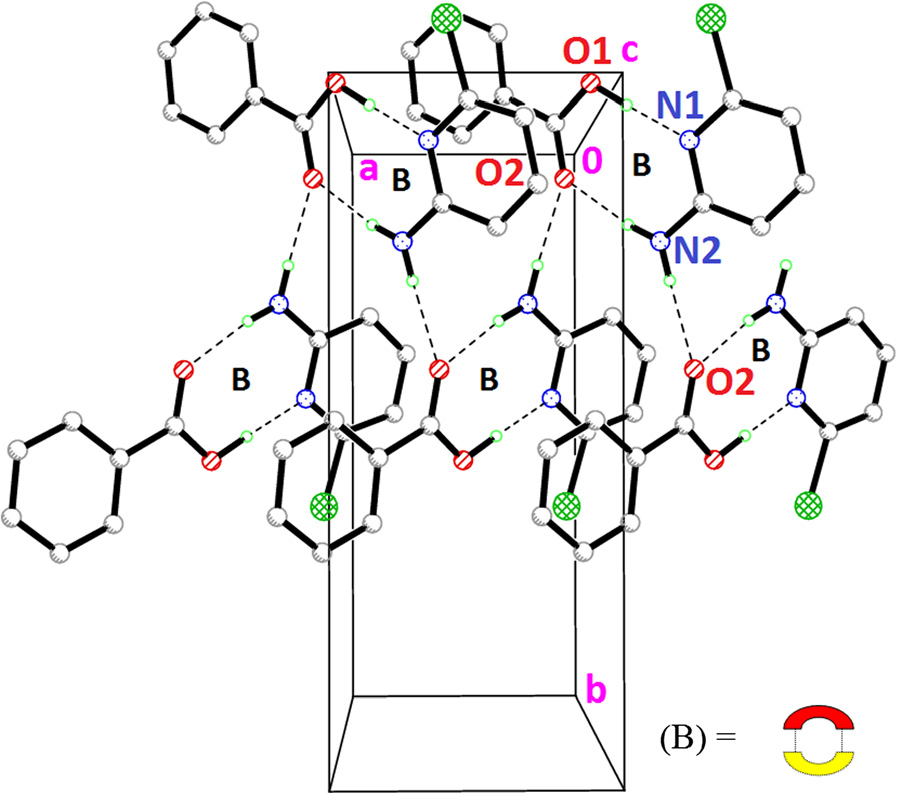
Hydrogen bonding patterns in compound (II), (B) represents heterosynthon.

**Figure 5:**
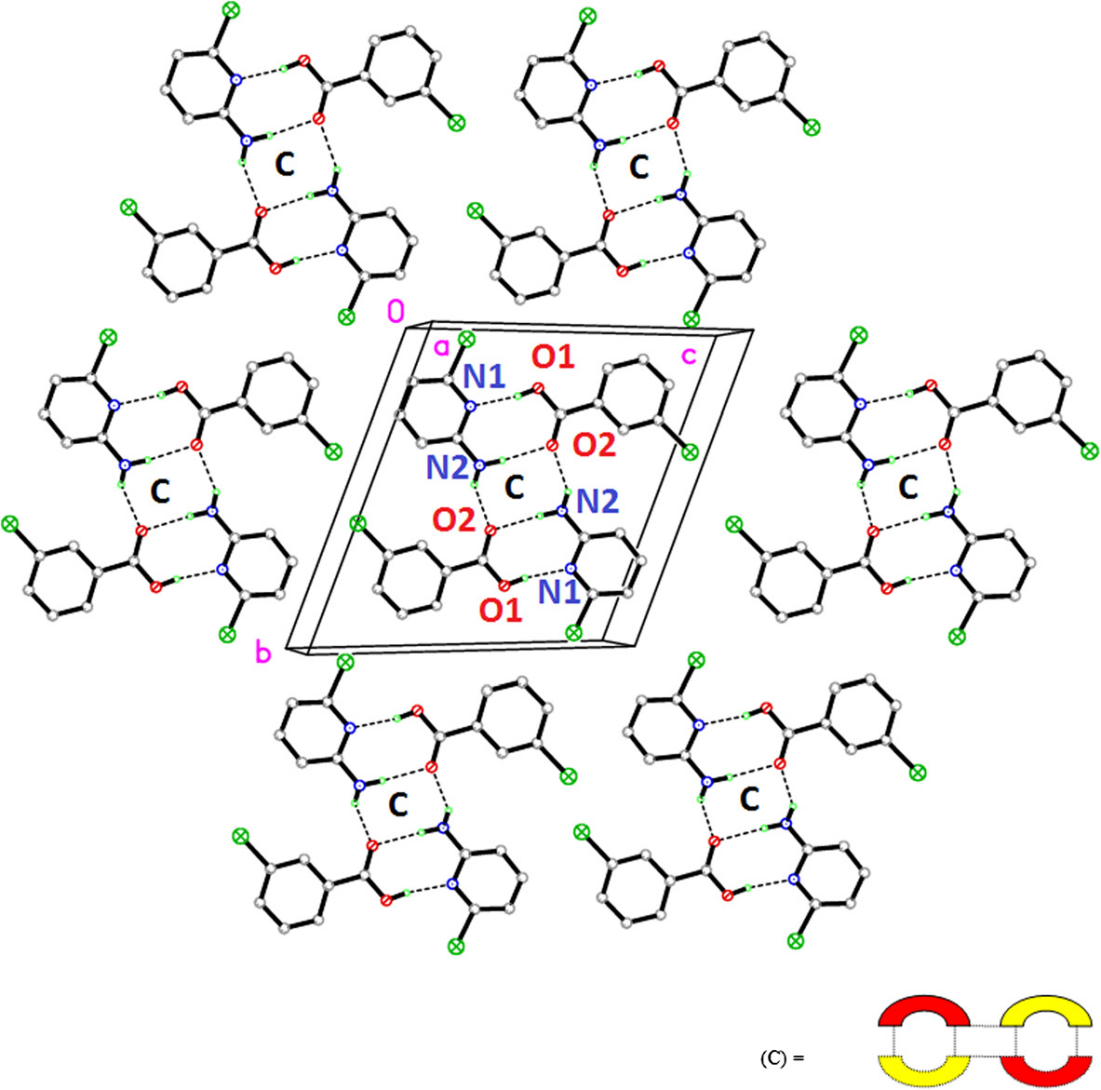
View of the complementary DADA (C) arrays of quadruple hydrogen bonding patterns in compound (IV).

**Figure 6:**
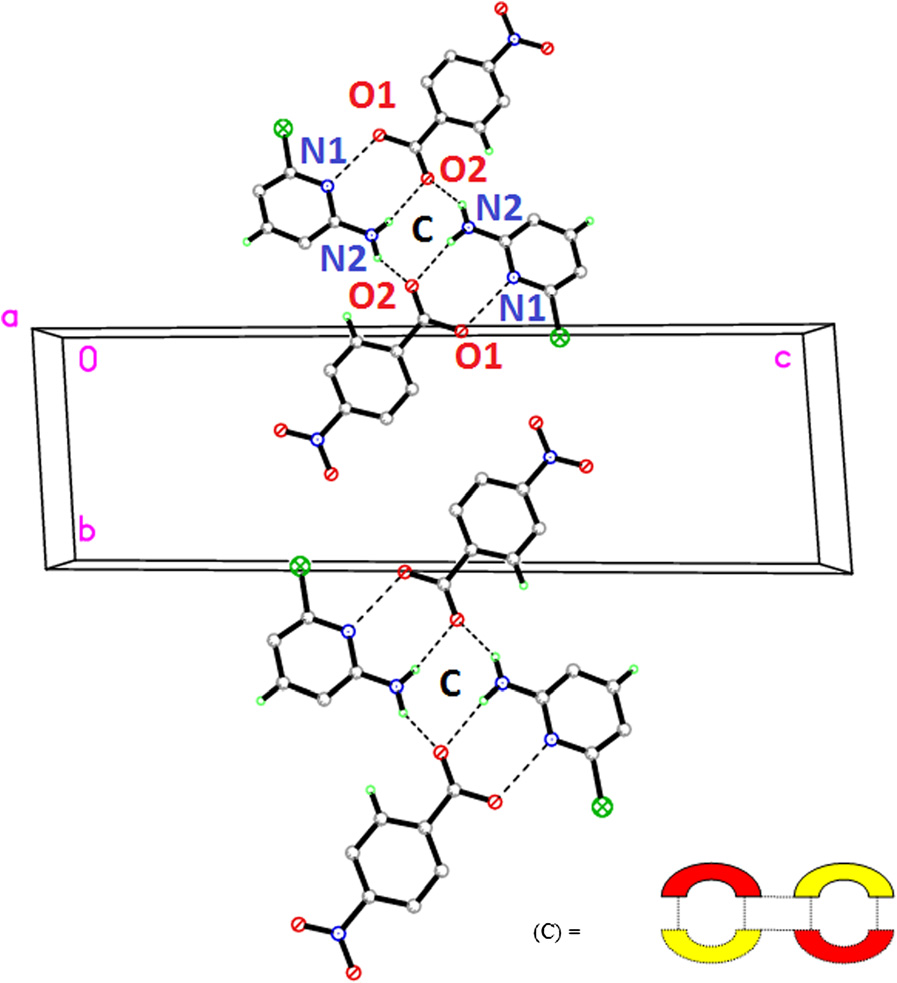
**Packing diagram for compound (V) (viewed along *a-*axis).** DADA (C) arrays are connected through N–H · · · O hydrogen bonds.

Scheme 1 illustrates all three types of hydrogen-bonded synthons observed in this study. In all compounds **(II-V)** except compound **(I)**, the carboxylic group of the respective acids (benzoic acid , 2-aminobenzoic acid, 3-chlorobenzoic acid and 4-nitrobenzoic acid) interacts with pyridine molecule in a linear fashion through N**---**H · · · O and O**---**H · · · N hydrogen bonds to form cyclic hydrogen-bonded motifs (heterosynthon). This can be designated by the graph-set notation $R_{2}^{2}\left(8\right)$. The motif has also been observed in a closely related 2-aminopyridine carboxylic acid co-crystal [17], and it is one of the 24 most frequently observed motifs in organic crystal structures [18]. In the crystal structure of compound **(I)**, the 2-amino-6-chloropyridine molecules are linked together into a centrosymmetric dimer *via* a pair of N2**---**H1N2 · · · N2 [1-*x*, -1/2 + *y*, 1/2-*z*; 155° & 3.3260 (19) Å] hydrogen bonds (homosynthon), involving the 2-amino group and the atom N1 of pyridine ring, to form an eight-membered hydrogen-bonded ring with a graph-set motif $R_{2}^{2}\left(8\right)$. These dimers are further interconnected by another N2**---**H2N2 · · · N1 [1-*x*, -*y*, 1-*z*; 173° & 3.1119(16) Å] and C4**---**H4A · · · Cl1 [*x*,-1/2-*y*,-1/2 + *z*; 144° & 3.6227(14) Å] hydrogen bonds, forming sheets parallel to the *bc* plane (Figure 2). In compound **(III)**, the torsion angle C3—C2—C1—O1 = -173.1 (2)° clearly shows the co-planarity of the carboxyl group and the benzene ring. An intramolecular N**---**H · · · O [129° & 2.686(6) Å] hydrogen bond is observed between the carboxylate oxygen (O2) and the amino N atom and thereby forming a characteristic *S*(6)-type motif as shown in Figure 3. When the donor and acceptor are in closeness on the same molecule, equilibrium may exist between closed conformations in which an intramolecular hydrogen bond is formed, creating a temporary ring system. Judging from the competition between inter and intramolecular hydrogen bonding, it is concluded that intramolecular hydrogen bonds are preferred when five or six-membered conjugated rings are formed. A pioneering statistical analysis of intramolecular hydrogen bonds in the Cambridge Structural Database (CSD) was performed by Bilton [19]. This analysis had studied 50 intramolecular hydrogen bond topologies and their tendency of formation within small molecule crystal structures. In compound **(II)**, intermolecular N2--H2N2 · · · O2 [1/2 + *x*, 3/2-*y*, 2-*z*; 172° & 2.905(2) Å] hydrogen bonds linked the heterosynthons into zig-zag chains (Figure 4). Whereas in compounds **(IV)** and **(V)**, these heterosynthons are centrosymmetrically paired *via* N**–**H · · · O hydrogen bonds, forming a complementary DADA [D = donor and A = acceptor] array of quadruple hydrogen bonds, with graph-set notation $R_{2}^{3}\left(8\right)$, $R_{2}^{2}\left(8\right)$ and $R_{2}^{3}\left(8\right)$, as shown in Figures 5 and 6. This type of DADA array has already been observed in many TMP-carboxylate complexes [16]. The DADA array of hydrogen-bonding motif can be represented in the form of three merged rings of $R_{2}^{3}\left(8\right)$, $R_{2}^{2}\left(8\right)$ and $R_{2}^{3}\left(8\right)$, in sequence, by using graph set notation. In **(V)**, the DADA arrays are further connected *via* C3A**---**H3AA · · · O3B [1 + *x*, *y*, *z*; 128° & 3.157(4) Å] hydrogen bonds, involving one of the carboxylate oxygen atoms (O3B) and one of the hydrogen atoms (H3AA) attached to C3A carbon atom, forming two-dimensional networks parallel to the *bc* plane.

## Conclusions

In this article, 2-amino-6-chloropyridine and its four co-crystals of benzoic acid derivatives were structurally characterized. It was observed that homosynthon was presented in crystal structure **(I)**, whereas carboxylic acid…pyridine heterosynthon were formed in all four co-crystals structures **(II)-(V)**. DADA arrays were observed in the crystal structures of **(IV)** and **(V)**. These DADA arrays have been observed in many 2,4-diaminopyrimidine carboxylate complexes as this motif is a potentially recurring synthon. Common laboratory analytical tools such as ^1^H NMR, ^13^CNMR, IR, photoluminescence, TEM analysis and XRD were used to understand the supramolecular architectures and to confirm the formation of the co-crystals. All co-crystals display photoluminescence in the solid state. The emission colours of the AMPY-BA derivatives-based building modules are significantly influenced by their incorporation of co-formers into the co-crystals.

## Experimental

### Materials and methods

2-Amino-6-chloropyridine (AMPY) **(I)** was used in this study. AMPY was reacted with a series of benzoic acid and its derivatives to form the following co-crystals: AMPY…BA **(II)**, AMPY…2ABA **(III)**, AMPY…3CLBA **(IV)** and AMPY…4NBA **(V)**.

### Synthesis of (I-V)

Hot methanol solution of 2-amino-6-chloropyridine (AMPY) **(I)** (57 mg, Aldrich) was warmed over a heating magnetic stirrer for 5 minutes. The resulting solution was allowed to cool slowly at room temperature. Crystals of the compound **(I)** appeared from the mother liquor after a few days. Compounds **(II-V)** were prepared by the mixing of hot methanolic solutions of AMPY (Sigma Aldrich, Malaysia) and the corresponding benzoic acid and its derivatives [2-amino benzoic acid, 3-chlorobenzoic acid and 4-nitrobenzoic acid (Sigma Aldrich, Malaysia) in a 1:1 molar ratio. The resultant mixtures were warmed over a water bath at 80˚C for 20 min, allowed to cool slowly and kept at room temperature for crystallization. After a few days, crystals of **(II-V)** were obtained.

### Transmission Fourier Transform Infrared (FTIR) spectroscopy

Transmission FTIR spectra were recorded on a PERKIN ELMER SPECTRUM GX (Perkin- Elmer Instruments LLC, Shelton, CT, USA). The KBr sample disk was scanned with a scan number of 8 from 400 to 4000 cm^-1^ having a resolution of 4 cm^-1^.

### ^1^H and ^13^C NMR spectroscopy

^1^H-NMR and ^13^C-NMR spectra were recorded at 400 MHz, in DMSO-d_6_, on Fourier transform Bruker spectrometer. The chemical shifts are reported in part per million (ppm) downfield from internal tetramethylsilane (TMS) (chemical shift in δ values). The spectroscopic details of NMR are summarized in Additional file 1: Table S2 and S3 (^†^ESI). The ^1^HNMR and ^13^CNMR spectra were shown in Additional file 1: Figures S2 and S3 (^†^ESI).

### Optical (OM) & Transmission Electron (TEM) Microscopes

An optical microscope (SZII; Olympus, Tokyo, Japan) equipped with a CCD camera (SSC-DC50A; SONY, Tokyo, Japan) was used to take images of crystal habit. Transmission Electron Micrographs (TEM) were obtained using a Philips TEM CM12 with an image analysis system. The specimen was prepared by depositing a drop of the alcholic solution of **I-V** suspension on the graphite grid sample holder and gently dried.

### Photoluminescence (PL)

PL spectra at room temperature of the samples were measured by Jobin Yvon HR 800 UV using 325 nm line of a He–Cd laser and Ar laser as the excitation source respectively. An analyzer was used to select the transverse-electric mode of the scattering light. Polarization-dependent PL spectra were performed at 15 K with a frequency-doubled Nd^+^-YAG laser at 532 nm as excitation source. The collected PL light was dispersed through a 0.5 m monochromator equipped with a 300 gr/mm grating and detected by an extended-InGaAs detector (detecting range: 0.5–1.1 eV). A linear polarizer was utilized to analyze the polarization of luminescence, and a depolarizer was placed in between the polarizer and monochromator to eliminate the response from the grating. In order to confirm the repeatability, the measurements were carried out for three times. Since the difference between the results was minimum (<0.1%), only one data from each measurement is presented for discussion.

### Powder X-ray Diffraction (XRPD)

XRPD diffractogram at 25˚C provided another piece of information for the identification and crystallinity of starting materials and co-crystals. Moreover, the powder diffraction patterns generated with the single-crystal data of compounds (I-V) using Mercury [20] matches accurately these experimental XRPD spectra measured using the D5000 powder diffractometer, thereby confirming the purity of the synthesized co-crystals. XRPD diffractograms were collected by SIEMENS D5000 DIFFRACTOMETER. The source of XRPD was CuKα (1.542 Å) and the diffractometer was operated at 40 kV and 30 mA. The X-ray was passed through a 1 mm slit and the signal a 1 mm slit, a nickel filter, and another 0.1 mm slit. The detector type was a scintillation counter. The scanning rate was set at 0.05° ranging from 5° to 35°. The quantity of sample used was around 20–30 mg.

### Single-Crystal X-ray data collection and structure determinations

Compounds **(I-V)** were examined under a microscope, and suitable single crystals were selected for X-ray analysis. Data were collected on a Bruker APEX2 CCD diffractometer with monochromatized MoKα radiation (*λ =* 0.71073 Å) equipped with an Oxford Cryo-system Cobra low-temperature attachment. Data for **(I-V)** were collected at 100 K. Lattice parameters were determined from least-squares analysis, and reflection data were integrated using the program SAINT. Lorentz and polarization corrections were applied for diffracted reflections. In addition, the data were corrected for absorption using SADABS. Structures were solved by direct methods and refined by full-matrix least-squares based on *F*^2^ using SHELXTL. Molecular graphics: SHELXTL, software used to prepare material for publication: SHELXTL and PLATON [21]. N- and O- bound hydrogen atoms were located from the difference Fourier map, and were refined with a riding model with *U*_iso_(H) = 1.2 or 1.5 *U*_eq_(N, O). The remaining hydrogen atoms in all the compounds **(I-V)** were positioned geometrically and refined as riding on their parent atoms, with *U*_*iso*_(H) = 1.2 *U*_eq_(C). Crystallographic data for compounds **(I-V)** are presented in Table 1, whereas hydrogen bond geometries are listed in Table 2.

### Supplementary materials

These data (CCDC 806013 **(I)**, CCDC 806014 **(II)**, CCDC 806010 **(III)**, CCDC 806011 **(IV)** and CCDC 806012 **(V)**) can be obtained free of charge at http://www.ccdc.cam.ac.uk/conts/retrieving.html/ or from the Cambridge Crystallographic Data Centre (CCDC), 12 Union Road, Cambridge CB2 IEZ, UK; fax: +44(0) 1223–336033; e-mail: deposit@ccdc.cam.ac.uk.

^†^ESI: Electronic Supporting Information.

## Competing interests

The authors declare that they have no competing interests.

## Authors’ contributions

MH carried out the synthesis and performed the IR and ^1^H NMR characterization. WSL, CKQ and HKF were involved in the single crystal X-ray data collection and elucidate the hydrogen bonding and their crystal packing patterns. MH was involved in the drafting of the manuscript. All authors read and approved the final manuscript.

## Supplementary Material

Additional file 1 Electronic Supporting Information (ESI). Click here for file
