# Supplementary material for: Investigation of supramolecular synthons and structural characterisation of aminopyridine-carboxylic acid derivatives
Source: Chem Cent J. 2014 May 6;8:31. doi: 10.1186/1752-153X-8-31 (PMC4032391; doi:10.1186/1752-153X-8-31)
Supplement: Additional file 1 — Electronic Supporting Information (ESI). [file 1752-153X-8-31-S1.docx]

**Electronic Supporting Information (ESI)**

**IR spectroscopy** is a well-organized method to determine the geometric structure of the molecules, and has been used widely in studying the structural consequences, such as in plane or out-of-plane vibrations. Herein, we have discussed the vibrational studies of substituted 2-aminopyridine (AMPY) with benzoic acid derivatives. The adduct formation is due to very high intermolecular hydrogen bonding forces that exist between the AMPY and the carbonyl group of benzoic acid derivatives. Hence, they were pulled towards each other to form a very strong adduct. Excepting for compound **(I)**, the remaining compounds **(II-V)** have the stretches near 1850 and 1650 cm^-1^ which are characteristics of intermolecular O**---**H···N hydrogen bonds that can only come about if the two reagents form hetero supramolecularsynthons (B). The vibrational spectra also suggest that the resulting products, in each case, can be classified as molecular co-crystals and not as organic salts. Figure S1 shows the FTIR spectrum recorded in the range 400-4000 cm^-1^ at room temperature. The assignment of FTIR data of the all co-crystals **(I-V)** is given in Table S1, which confirms the presence of different functional groups of the products.

***C-H vibrations:***

The stretching vibrations of di-substituted pyridine with benzoic acid adducts [1] fall in the range of 3100-3000 cm^-1^. In this region, the bands are not affected significantly by the nature of substituent. In **(I-V),** the molecules give rise to C-H stretching, C-H in-plane bending vibrations and C-H out-of-plane bending vibrations. The aromatic C-H stretches in the region 3081-3052 cm^-1^ are in agreement with experimental assignment [2] 3093-3026 cm^-1^. The C-H out-of-plane bending falls in the FTIR values of 1006-827 cm^-1^. The C-H in-plane bending vibrations are assigned to the region 1221-1110 cm^-1^ even though they are found to be tainted by C-O stretching and O-H in-plane bending in the range as commented in literature [3], while the experimental observations are at 1255-1103 cm^-1^.

***C-C vibrations:***

The aromatic ring stretching modes appear in very thin spectral ranges 1625-1570 and 1470-1425 cm^-1^, respectively, in di-substituted benzene and pyridine derivatives [4]_._ The real positions are determined not so much by the nature but by the position of the substituent around the ring. The bands observed at 1604-1417 cm^-1^ranges are identified as C-C stretching vibrations. The C-C aromatic stretch, known as semi-circle stretching [5], predicted at 1569 cm^-1^ is also in excellent agreement with experimental observations of 1581-1557 cm^-1^ in FTIR spectrum.

***C=N, C-N vibrations:***

The recognition of C-N vibrations is a very difficult task, since the mixing of several bands are possible in this region. Silverstein [5] assigned C-N stretching absorption in the region 1382-1266 cm^-1^ for aromatic amines. In benzamide, the band observed at 1368 cm^-1^ is assigned to be due to C-N stretching [5]. In benzotrizole, the C-N stretching bands are found to be present at 1382 and 1307 cm^-1^. In the present work, the band observed at 1490-1443 cm^-1^ and 1372-1357 cm^-1^ in the FTIR spectrum have been assigned to C=N, C-N stretching vibrations, respectively.

***C-NH_2_ vibrations:***

The scaled -NH_2_ symmetric and asymmetric stretches in the range 3555-3446 cm^-1^ [5] is in agreement with the experimental value of 3480-3272 cm^-1^. The -NH_2_ scissoring vibration is at 1600 cm^-1^ [5] is also in very good agreement with the recorded FTIR value of 1640-1629 cm^-1^. FTIR stretching mode with 1311-1331 cm^-1^ corresponding to C-NH_2_ moiety was calculated to be 1298 cm^-1^. The C-NH_2_ in-plane bending vibrations at 476-455 cm^-1^, are also in good agreement with the experimental data.

***C-NO_2_ vibrations:***

The substituted nitro group at the fourth position of the benzoic acid molecule would increase the C-NO_2_ stretching vibrations in addition to the internal vibrations. The very strong FTIR band appearing at 1602 cm^-1^ is assigned to C-NO_2_ stretching vibrations. In the vibrational spectra of 2-chloro-3-nitropyridine and 2-methyl-8-nitroquninoline [6], various internal vibrations of the nitro group were recognized. In the present study, a very strong FTIR band is observed at 1565-1527 cm^-1^ and a FTIR band at 1357-1311 cm^-1^ are assigned to the asymmetric and symmetric stretching of the NO_2_ group respectively. NO_2_ in-plane deformation and NO_2_ wagging vibrations appear in the 870-850 and 625-485 cm^-1^ regions, respectively, in various substituted nitrobenzenes [7] and nitropyridines [8]. The frequency at 878-850 cm^-1^ in FTIR is allocated to NO_2_ bend mode whereas the frequency at 544-516 cm^-1^ was assigned to NO_2_ wagging [9]. NO_2_ rocking vibrations have been observed in the locality of 510-472 cm^-1^. The frequency at 472 cm^-1^ region is a suitable choice for NO_2_ rocking vibrations. These assignments are in good agreement with the former reports.

***C-Cl vibrations:***

The vibrations belonging to the bonds between the ring and halogen atoms are essential to be discussed here since mixing of vibrations are possible due to the lowering of the molecular symmetry and the presence of heavy atoms on the periphery of the molecule. The assignment of C-Cl stretching and deformation vibrations has been made by comparison with similar molecules, such as, *p*-bromophenol and the halogen substituted benzene derivatives [10]. Mooney [7], assigned vibrations of C-X group (X= Cl, Br, I) in the frequency range of 1129-480 cm^-1^. Compounds with more than one chlorine atom reveal very strong bands due to the asymmetric and symmetric stretching modes. Vibrational coupling with other groups may result in a shift in the absorption to frequency as high as 840 cm^-1^. For simple organic chlorine compounds, C-Cl absorptions are in the region of 747-708 cm^-1^ whereas for the trans and gauche forms, [5] they are near 650 cm^-1^.

***COOH vibrations:***

Bands due to O-H stretching vibrations, υ(OH), are much more stronger in IR spectra. When carboxylic groups form hydrogen bonding, the result is a broad band centered at 3225-3377cm^-1^, that superimposes onto the υ(C-H) bands. The most characteristic aspect of carboxylic group is a single band usually in the range 1800-1700 cm^-1^. This band is due to the C=O stretching vibration, υ(C=O). The band appearing at 1649-1725 cm^-1^ is assigned as C=O stretching vibration in FTIR spectrum. Another two typical carboxylic group vibrations are: C-O stretching υ(C-O), and in-plane C-O-H bending, υ(C-OH). They are expected in the 1450-1150 cm^-1^ region depending on whether monomeric, dimeric or other hydrogen bonded types are present [5, 10].

**^1^H and ^13^C NMR spectra**

The ^1^H and ^13^CNMR spectra of all the co-crystals **(I-V**) were carried out in DMSO-d_6_ at room temperature using TMS as internal standard and are shown in Figures S2 & S3. The signals in the 6.47-8.47 ppm range are usual for hydrogen’s attached to an aromatic (benzene) ring. The hydrogen of the carboxylic acid (COOH) produces a broad signal at 11.00-11.50 ppm. ^1^H NMR spectrum characterized by the presence of broad band in the range of δ = 7.00-7.74 are assigned to the NH_2_ groups. In the ^13^C spectrum [11, 12], the signals of the aromatic carbons at approx. 106.5-142.0 and 159.0-162.5 ppm are assigned for (=C-N-) carbons, respectively. The (=C-H) groups appear at δ =119.90 and 120.16 ppm, respectively. Furthermore, the (C=O) groups resonance is at δ=162.0-165.0 ppm. Other characteristic chemical shifts are listed in Tables S2 & S3.

**Photoluminescence properties**

Luminescent compounds are of vast current interest because of their various applications in chemical sensors, photochemistry and electroluminescent displays. The luminescence of aromatic compounds is caused by the delocalization of the conjugated π electrons on the aromatic ring. The photoluminescence (PL) spectrum is a valuable method to confirm quantity of the LEDs [13]. The solid-state PL emission spectraof AMPY **(I)**, and the stoichiometry co-crystals of AMPY…BA **(II),** AMPY…2ABA **(III),** AMPY…3CLBA **(IV)** and AMPY…4NBA (2:2) **(V)** were studied at room temperature. The emission spectra were recorded between the region of 300-1000 nm. Figure S4 shows that the free ligand AMPY displays luminescence with emission maxima at 390 nm and their co-crystals of **(II-V)** exhibit emission maxima at 550 nm upon excitation at 300 nm. It can be reputed that these peaks should be assigned to the π*→π and n→π* transitions on the basic of the emission of acid and base components. The observed variation is perhaps due to the presence of multiform hydrogen bonds, π···π interactions and C---H···π interactions in the crystal packing. The usual pyridine–carboxylic acid interactions primary to the formation of O---H···N and N---H···O hydrogen bonds can exhibit emission maxima at 587 to 565 nm [14]. This study also demonstrates the reality that the pyridyl ring plays an important and effective role in the formation of co-crystals. The difference in molecular packing, conformations, hydrogen bonding interactions and solvent molecules involved could also affect the emission properties of the co-crystals in the solid state.

**Powder diffraction**

Powder diffraction patterns obtained for AMPY **(I)** with aromatic carboxylic acid derivatives, such as BA, 2ABA, 3CLBA, 4NBA and the stoichiometric 1:1 co-crystals of AMPY...BA **(II)**, AMPY...2ABA **(III)**, AMPY...3CLBA **(IV)** and AMPY...4NBA **(V)** components are shown in Figures S5-8.

***Compound II:*** XRPD patterns obtained for BA, AMPY, and the stoichiometric 1:1 AMPY…BA components are shown in Figure S5. The diffraction patterns of the three materials were found to be very different, with the co-crystal of compound **II** exhibiting characteristic peaks at 10.85, 13.43, 21.49, 22.66, 23.92, 27.59, 32.13 and 40.25 deg 2θ, and BA exhibits characteristic peaks at 16.40, 17.36, 24.47, 26.18, 27.74 and 30.31 deg 2θ, whereas AMPY exhibits characteristic peaks at 15.84 and 24.12 deg 2θ.

***Compound III:*** XRPD patterns obtained for 2ABA, AMPY, and the stoichiometric 1:1 AMPY…2ABA components are shown in Figure S6. The diffraction patterns of the three materials were found to be very different, with the co-crystal of compound **III** exhibiting characteristic peaks at 15.94, 18.82, 24.47, 30.3 and 42.22 deg 2θ, and 2ABA exhibits characteristic peaks at 16.6, 19.23, 24.48, 28.05 and 30.5 deg 2θ, whereas AMPY exhibits characteristic peaks at 15.84 and 24.12 deg 2θ.

***Compound IV:*** XRPD patterns obtained for 3CLBA, AMPY, and the stoichiometric 1:1 AMPY…3CLBA components are shown in Figure S7. The diffraction patterns of the three materials were found to be very different, with the co-crystal of compound **IV** exhibiting characteristic peaks at 13.83, 16.25, 17.81, 23.91, 26.43, 29.41, 34.70, 41.36 and 50.84 deg 2θ, and 3CLBA exhibits characteristic peaks at 13.68, 18.52, 23.46, 27.84, 33.54, 36.6 and 45.34 deg 2θ, whereas AMPY exhibits characteristic peaks at 15.84 and 24.12 deg 2θ.

***Compound V:*** XRPD patterns obtained for 4NBA, AMPY, and the stoichiometric 1:1 AMPY…4NBA components are shown in Figure S8. The diffraction patterns of the three materials were found to be very different, with the co-crystal of compound **V** exhibiting characteristic peaks at 21.04, 23.46, 24.87, 26.69, 27.19, 27.80, 28.71, 29.11, 29.81 and 39.10 deg 2θ, and 4NBA exhibits characteristic peaks at 14.33, 16.30, 24.57, 28.70, 30.08 and 41.96 deg 2θ, whereas AMPY exhibits characteristic peaks at 15.84 and 24.12 deg 2θ.

The XRPD pattern of the AMPY…BA, AMPY…2ABA, AMPY…3CLBA and AMPY…4NBA differed from those of the constituents, confirming the formation of a new complex phase. In addition, the powder diffraction patterns generated with the single-crystal data of compounds (**I-V**) using Mercury [15] matches accurately these experimental XRPD spectra measured using the D5000 powder diffractometer, thereby confirming the purity of the synthesized co-crystals.

**TEM:** Crystal excellence (crystal shape, crystal surface, and crystal defects) is one of the major factors playing a vital role for safer storage, transport, and handling while maintaining their performance. Transmission electron microscopy (TEM) has long been used in materials science as a powerful analytical tool[36] for crystal quality. The co-crystals morphologies were analyzed by TEM. Figure S9 reveals the morphological differences between the AMPY, BA-derivatives and the synthesized AMPY…BA-derivatives co-crystals.

**Table S1 - Infrared spectral (cm^-1^) data of the compounds (I-V)**

| Compound | υ (C-H)  aromatic | υ (C=C) | υ (C-N) | υ (C=N) | υ (N-H) | υ (C=O) | υ (O-H) | υ (C-Cl) |
| --- | --- | --- | --- | --- | --- | --- | --- | --- |
| AMPY | 3072 | 1417 | 1357 | 1487 | 3272 | - | - | 747 |
| AMPY…BA | 3064 | 1422 | 1363 | 1483 | 3439 | 1649 | 3343 | 721 |
| AMPY…2ABA | 3081 | 1432 | 1372 | 1467 | 3480 | 1710 | 3377 | 708 |
| AMPY…3CLBA | 3073 | 1604 | 1365 | 1443 | 3467 | 1725 | 3225 | 735 |
| AMPY…4NBA | 3052 | 1421 | 1363 | 1490 | 3360 | 1725 | 3360 | 722 |

**Table S2 - ^1^H NMR spectral data (δ, ppm) of the AMPY (I) and with co-crystals (II-V)**

| Compound | (C-H)- aromatic | (N-H) | (O-H) |
| --- | --- | --- | --- |
| AMPY | 6.57-6.70 | 7.72 | - |
| AMPY…BA | 6.47-8.21 | 7.71 | 11.0 |
| AMPY…2ABA | 6.79-7.64 | 7.00 | 11.5 |
| AMPY…3CLBA | 6.76-8.09 | 7.74 | 11.5 |
| AMPY…4NBA | 6.76-8.47 | 7.74 | 11.0 |

**Table S3 - 13C NMR spectral data (δ, ppm) of the AMPY (I) and with cocrystals (II-V)**

| Compound | **Aromatic carbons** | **(=C-N)** | **(C=O)** | **(C-Cl)** |
| --- | --- | --- | --- | --- |
| AMPY | 106.5-112.5 | 159.5 | - | 150.5 |
| AMPY…BA | 107.5-140.0 | 160.5 | 164.5 | 152.5 |
| AMPY…2ABA | 108.0-138.5 | 162.5 | 162.0 | 154.0 |
| AMPY…3CLBA | 107.5-140.0 | 159.0 | 162.0 | 149.5 |
| AMPY…4NBA | 108.0-142.5 | 160.5 | 165.0 | 152.0 |

**References**

1. Rastogi K, Palafox MA, Tanwar RP, Mittal L: **3,5-Difluorobenzonitrile: ab initio calculations, FTIR and Raman spectra.** *Spectrochim Acta A* 2002,**58**:1987-2004
2. Varasanyi G: *Assignments For Vibrational Spectra of Seven HundredBenzene Derivatives*, Vols I and II Adam Hilger 1974
3. Lee C, Yang W, Parr RG: **Development of the Colle-Salvetti correlation-energy formula into a functional of the electron density.** *Phys Rev B* 1988, **37**:785-789
4. Silverstein M, Clayton G, Basseler M, Morill C: *Spectrometric Identification of Organic Compounds*, Wiley New York 1981
5. Sing NP, Yadav RA: **Vibrational studies of trifluoromethyl benzene derivatives I: 2-amino-5-chloro and 2- amino-5-bromo benzotrifluorides.** *Ind J Phys B* 2001, **75(4):**347-355
6. Sarma M, Mishra MK: **Role of photolysis frequency in enhanced selectivity and yield for controlled bond breaking in HOD.** *J Phys Chem A* 2008, **112**:4895
7. Mooney EF: **The infrared spectra of chloro- and bromobenzene derivatives—I: Anisoles and phenetoles.** *Spectrochim Acta* 1963, **19**:877-887
8. Green JHS, Harrison DJ: **Vibrational spectra of benzene derivatives—X: Monosubstituted nitrobenzenes.** *Spectrochim Acta A* 1970, **26**:1925-1937
9. Berezin VI, Elkin MD: **Taking into Account Influence of Substitution on Force-Field of an Aromatic Ring.** *Opt Spectrosc* 1974, **37**:237-240
10. Varasanyi G: *Assignments For Vibrational Spectra of Seven Hundred Benzene Derivatives*, Vols I and II Adam Hilger 1974.
11. Özden S, Karatas H, Yıldız S, Göker H: **Synthesis and Potent Antimicrobial Activity of Some Novel 4-(5, 6-Dichloro-1H-benzimidazol-2-yl)-N-substituted Benzamides.** *Arch. Pharm Pharm Med Chem* 2004, **337**:556-562
12. Ha ST, Ong LK, Win YK, Koh TM, Yeap GY: **Synthesis of New Schiff Base: 4-[(Pyridin-3-ylmethylene)-amino]phenyldodecanoate.** Molbank 2008, **2008(3)**:M582
13. Shih PI, Chien CH, Wu FI, Shu CF: **A Novel Fluorene-Triphenylamine Hybrid That is a Highly Efficient Host Material for Blue-, Green-, and Red-Light-Emitting Electrophosphorescent Devices.** *Adv Funct Mater* 2007, **17**:3514-3520
14. Anthony SP, Varughese S, Draper SM: **Impact of molecular structure on intermolecular interactions and organic solid state luminescence in supramolecular systems.** *J Phy Org Chem* 2010, **23**:1074-1079
15. Macrae CF, Edgington PR, McCabe P, Pidcock E, Shields GP, Taylor R, Towler M, van de Streek J: **Mercury: visualization and analysis of crystal structures.** *J Appl Cryst* 2006, **39**:453-457

**
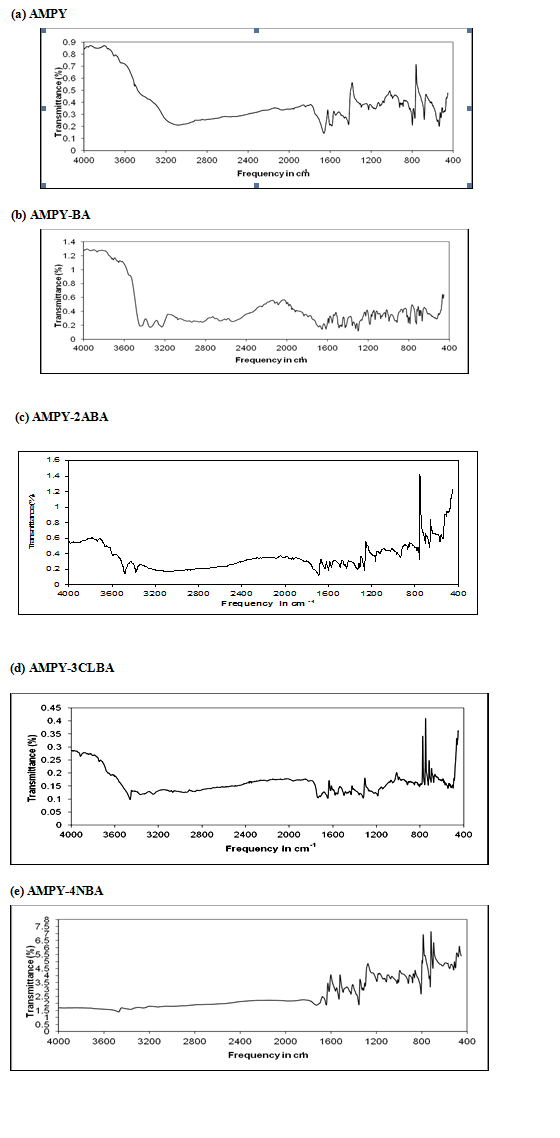
**

**Figure S1: Comparison of FTIR spectra. (a)** AMPY, **(b)** AMPY…BA, **(c)** AMPY…2ABA, **(d)** AMPY…3CLBA and **(e)** AMPY…4NBA.


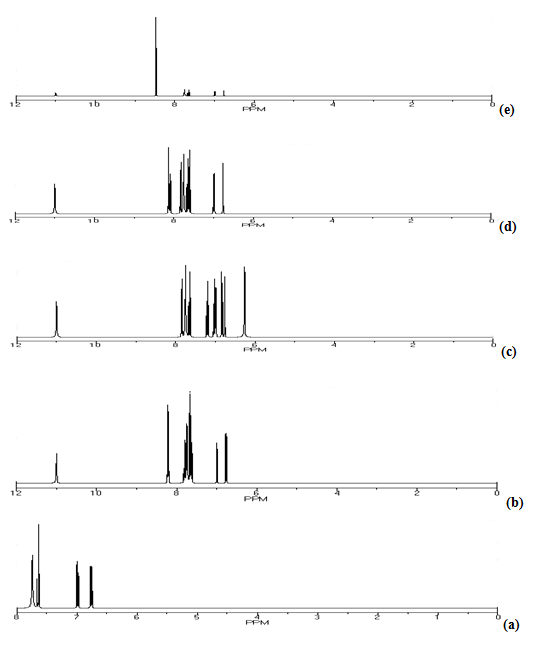


**Figure S2. Comparison of ^1^HNMR spectra. (a)** AMPY, **(b)** AMPY…BA, **(c)** AMPY…2ABA, **(d)** AMPY…3CLBA and **(e)** AMPY…4NBA in d6-DMSO solvent.

**
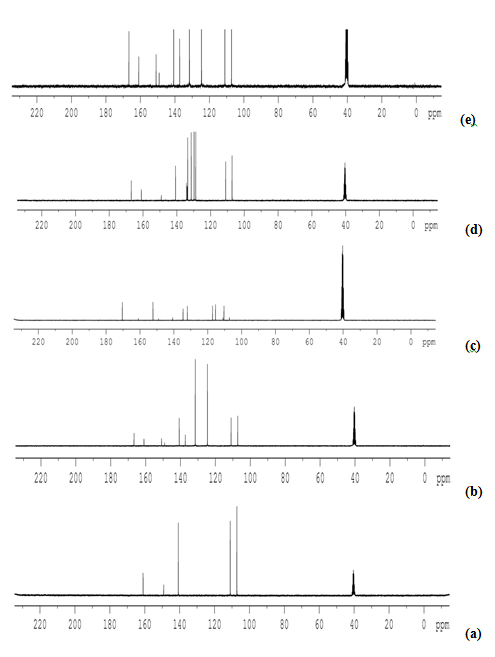
**

**Figure S3: Comparison of ^13^CNMR spectra. (a)** AMPY, **(b)** AMPY…BA, **(c)** AMPY…2ABA, **(d)** AMPY…3CLBA and **(e)** AMPY…4NBA in d6-DMSO solvent.


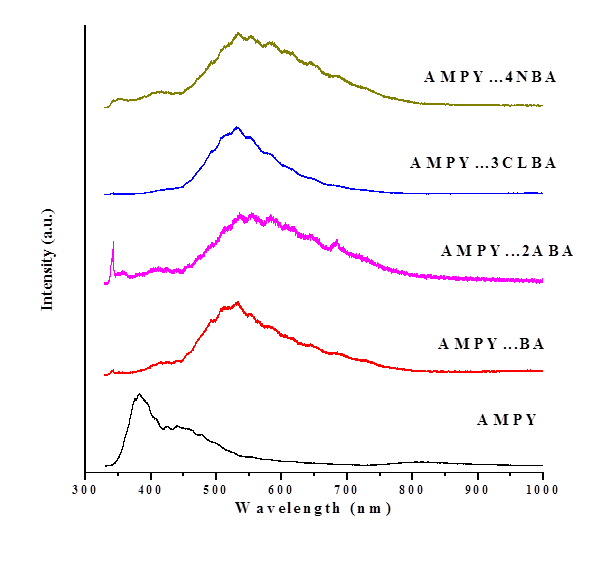


**Figure S4: Photoluminescences of ligands and co-crystals (I-V).**

**
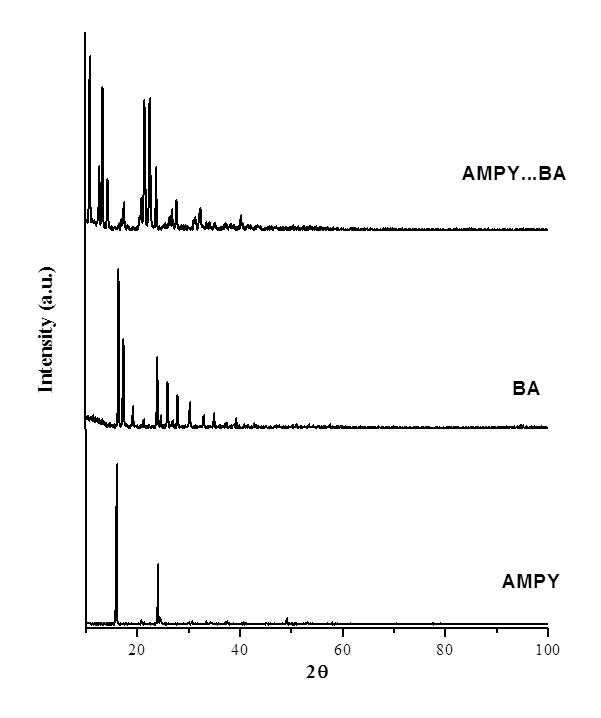
**

**Figure S5: Comparison of experimental Powder X-ray diffraction patterns. (a)** AMPY, **(b)** BA, and **(c)** the co-crystal for AMPY…BA.

**
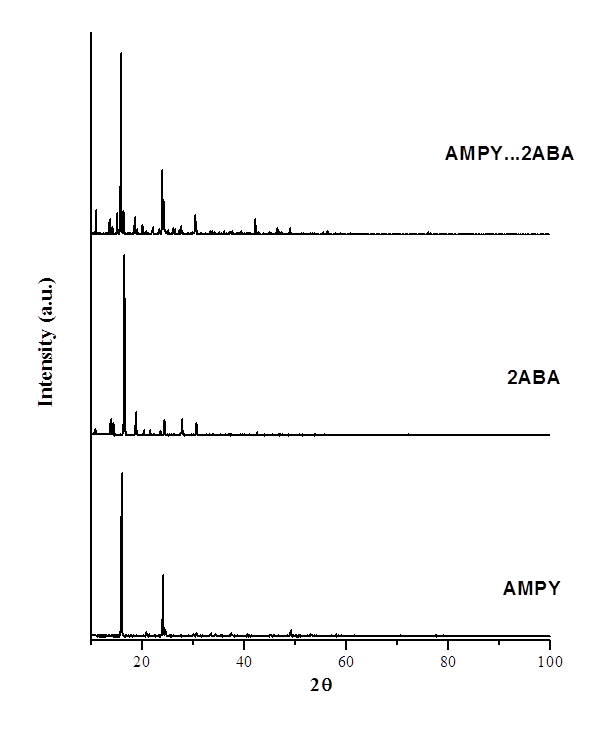
**

**Figure S6: Comparison of experimental Powder X-ray diffraction patterns. (a)** AMPY, **(b)** 2ABA, and **(c)** the co-crystal for AMPY…2ABA.

**
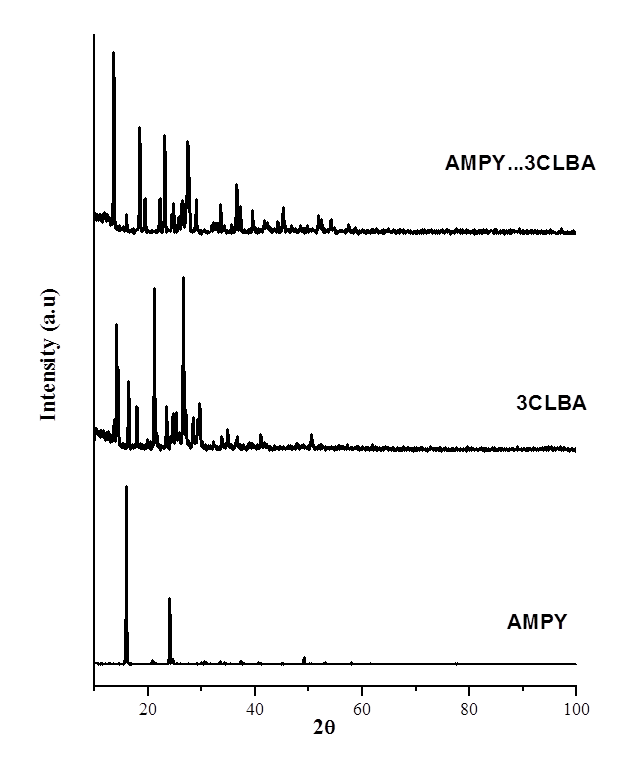
**

**Figure S7: Comparison of experimental Powder X-ray diffraction patterns. (a)** AMPY, **(b)** 3CLBA, and **(c)** the co-crystal for AMPY…3CLBA.

**
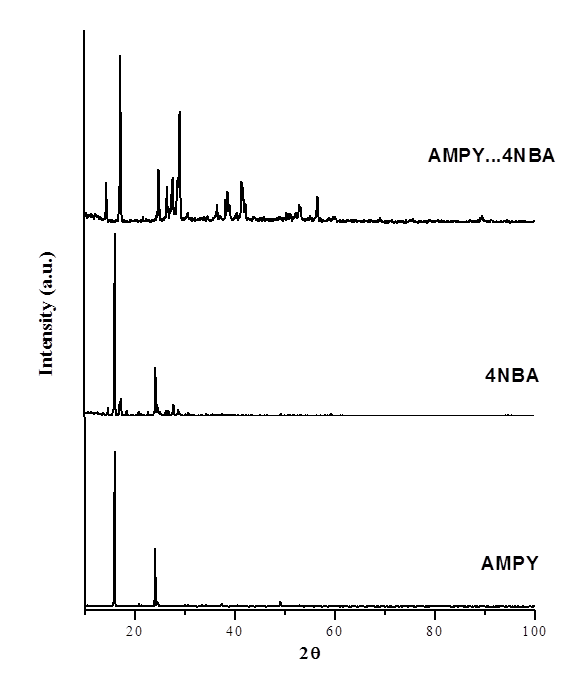
**

**Figure S8: Comparison of experimental Powder X-ray diffraction patterns. (a)** AMPY, **(b)** 4NBA, and **(c)** the co-crystal for AMPY…4NBA.


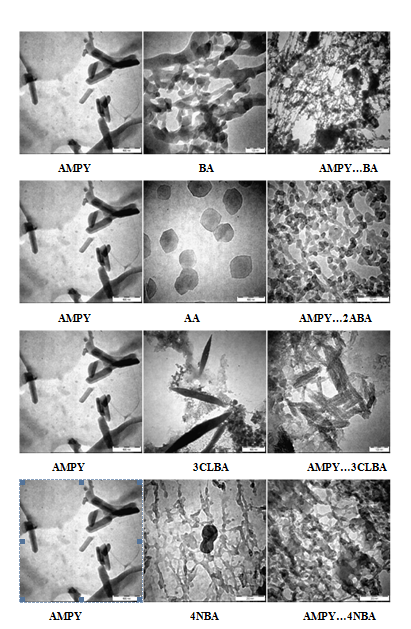


**Figure S9: TEM images of co-crystals of AMPY, BA-derivatives and AMPY…BA derivatives (co-crystals).**
